# The Southern African Centre for Infectious Disease Surveillance: A One Health Consortium

**DOI:** 10.3402/ehtj.v6i0.19958

**Published:** 2013-01-25

**Authors:** Mark M. Rweyemamu, Peter Mmbuji, Esron Karimuribo, Janusz Paweska, Dominic Kambarage, Luis Neves, Jean-Marie Kayembe, Aaron Mweene, Mecky Matee

**Affiliations:** 1 SACIDS at Sokoine University of Agriculture, Morogoro, Tanzania; 2 Department of Preventive Services, Ministry of Health and Social Welfare, Dar es Salaam, Tanzania; 3 National Institute for Communicable Diseases of the National Health Laboratory Services, Sandringham, Johannesburg, South Africa; 4 Faculty of Veterinary Medicine, Eduardo Mondlane University, Maputo, Mozambique; 5 School of Public Health, University of Kinshasa, Democratic Republic of Congo; 6 School of Veterinary Medicine, University of Zambia, Lusaka; 7 Department of Microbiology and Immunology, Muhimbili University for Health and Allied Sciences, Dar es Salaam, Tanzania

**Keywords:** SACIDS, SADC, One Health, mobile ICT, CORDS, wildlife-livestock-human interaction

## Abstract

Formed in 2008, the Southern African Centre for Infectious Disease Surveillance (SACIDS) is a One Health consortium of academic and research institutions involved with infectious diseases of humans and animals. Operating in partnership with world-renowned centres of research in industrialised countries, its mission is to harness innovations in science and technology for improving southern Africa's capacity to detect, identify, monitor (DIM) and manage the risk posed by infectious diseases of humans, animals, and ecosystems. The consortium's major capacity development activities include a series of One Health-based Master of Science (MSc) courses and a five-year DIM-driven research program. Additionally, SACIDS organized Africa's first One Health conference, in July 2011. This paper describes these and other major activities that SACIDS has undertaken to improve infectious disease surveillance across southern Africa. The paper also describes the role and collaboration of SACIDS with other national, regional and international consortia/networks that share a vision and interest in promoting novel approaches to infectious disease surveillance and outbreak response.

## Introduction

Founded in 2008, the Southern African Centre for Infectious Disease Surveillance (SACIDS) services the Southern African Development Community (SADC). Operating in partnership with world renowned research institutions in the United Kingdom, United States, and Asia, SACIDS (www.sacids.org) is a virtual center that links expertise and physical resources across institutions and health sectors with the goal of developing southern Africa's capacity for the cost-effective risk management of its regional infectious disease burden threat. It has become increasingly apparent that the most cost-effective strategy for addressing the high infectious disease burden and risk in southern Africa must be through the sharing of expertise and resources across institutions; through close collaboration between the human and animal health sectors; and, ideally, through an approach that is based on ecological systems, which in Africa often transcend administrative or national boundaries (1).

The underlying concepts for One Health have long been recognized. In the 19th century, Rudolf Virchow observed, “Between animal and human medicine there is no dividing line – nor should there be. The objective is different, but the experience obtained constitutes the basis of all medicine” (2). In the 1960's, Calvin Schwabe stated, “There is no difference between human and veterinary medicine. Both sciences share a common body of knowledge in anatomy, physiology, pathology, on the origins of diseases in all species” (3). However, only recently, largely through the risk of emerging infectious diseases, has the One Health paradigm come into sharp focus, with the dialogue shifting from one centered on the practice of One Health (“One Medicine”) to one centered on One Health as an outcome or goal (4, 5). Yet, still there is no universally agreed definition of One Health. At the 2007 American Veterinary Medical Association convention, Lonnie King described One Health as “a holistic systems approach to understanding health across all species” (6). He explained, “ It's a recognition that human and animal health are inextricably linked, and One Health is about how to promote, improve, and defend the health and well-being of all species, with the cooperation of physicians and veterinarians.” The AVMA describes One Health as a “collaborative effort of multiple disciplines working locally, nationally, and globally to attain optimal health for people, animals and our environment” (7). An arguably more comprehensive description is the European Commission definition: “the improvement of health and well-being through (i) the prevention of risks and the mitigation of effects of crises that originate at the interface between humans, animals and their various environments, and (ii) promoting a cross-sectoral, collaborative, ‘whole of society’ approach to health hazards, as a systemic change of perspective in the management of risks” (8).

In the absence of a single universally accepted definition of One Health, the trend is for practitioners to describe their own One Health focus, often reflecting the underlying driver or objective of their work. Given that infectious diseases drive the mission of SACIDS, the SACIDS focus on One Health seeks to apply the concept to the management of infectious disease risk. Accordingly, we have identified the SACIDS focus on One Health as: a collaborative effort between natural and social scientists to advance the understanding of interactions between humans, animals, and their environment in the endemic settings of southern Africa. While SACIDS's One Health focus broadly relates to definitions for One Health described by others (2–8), the SACIDS focus firmly reflects its own vision and mission. The vision of SACIDS is a southern African society protected from devastating infectious diseases affecting the health of humans, animals (i.e. both terrestrial and aquatic), and ecosystems (i.e., crop, fruit, and ornamental), thereby promoting livelihoods, socio-economic development (including market access), and the environment. The consortium's mission is to harness innovation in science and technology for improving southern Africa's capacity to detect, identify, and monitor infectious diseases of humans, animals, ecosystems and their interactions and to better manage the risk posed by them.

The need for a One Health approach is supported by the findings of several recent studies (9–15). Together, these studies have shown that about 60 percent of all infectious pathogens of humans originate from animals and that, over the last 25 to 30 years, some 70 to 75 percent of emerging infectious diseases in humans originated in animals. That trend is expected to continue in the future, as economic development, changes in habitation and farming systems, globalization of travel and trade, and climatic variations continue to fuel the emergence and spread of infectious diseases. Many of these animal-originating emerging diseases in humans are endemic to Africa (and Asia) and constitute a high risk for future marginalization of Africa; and could severely constrain human mobility and access to international markets for African animal and plant commodities. Reducing the risk posed by animal and human pathogens – and their interactions – requires more than an understanding of the diseases themselves. It also requires an understanding of the social context of disease. SACIDS researchers advocate that substantial advances in infectious disease prevention and management could be made not just by integrating research across health sectors (human, animal, ecosystem), but also across disciplines (natural and social science) (16).

SADC is geographically and culturally linked to the five-country East African Community (EAC): Burundi, Rwanda, Uganda, Kenya, and Tanzania. Together, the two regional economic communities (RECs) share not only a vision for inter-regional free trade, but also an abundance of wildlife animals in their savannah and forest ecosystems and an intense wildlife-livestock-human dynamic (17). Thus, some SACIDS activities are conducted in collaboration with the East African Integrated Disease Surveillance Network (EAIDSNet) (18).

## Governance

The headquarters of SACIDS is located at the Sokoine University of Agriculture (SUA), Morogoro, Tanzania. SACIDS operates as a non-profit inter-institutional consortium through the legal framework of the SACIDS secretariat host (i.e., SUA) and member institutions. At the national level, several virtual centers for infectious diseases have been formed. Collectively, these National Centres for Infectious Disease Surveillance (NatCIDS) form the core of SACIDS (see Table 1). The underlying concept of the consortium's governance is equitable representation of the human and animal health sectors at both the national and regional levels in order to ensure effective inter-sectoral collaboration. SACIDS also operates in partnership with world-renowned centres of research in several industrialised countries, especially the University of London Colleges, United Kingdom. For a complete list of partnerships, see Table 1.

**Table 1:** Participants in the SACIDS Consortium

Partnership Category	Partners		
**Southern African pioneer partners and constituent members of SACIDS by agreement**	*Partner*	*National Coordinator*	*Constituent Members*
	Tanzanian National Consortium	Professor Mecky Matee, Head Dept Microbiology, Muhimbili University of Health and Allied Sciences, MUHAS, Dar es Salaam, Tanzania	The National Institute for Medical Research (NIMR) Ifakara Health Research & Development Centre, Tanzania The Muhimbili University of Health and Allied Sciences (MUHAS) The Faculty of Veterinary Medicine, Sokoine University (FVM-SUA) The Central Veterinary Laboratory (CVL) The Tanzania Wildlife Research Institute (TAWIRI) The Institute of Resource Assessment (IRA), University of Dar es Salaam
	Democratic Republic of Congo (DRC) National Consortium	Professor Jean-Marie Kayembe Ntumba, Associate Dean, Faculty of Medicine, Institute of Public Health Kinshasa, DRC	The Institute of Public Health of the Faculty of Medicine of the University of Kinshasa The Faculty of Veterinary Medicine of the University of Lubumbashi National Institute for Biomedical Research (INRB) The Central Veterinary Laboratory in Kinshasa National Institute for Nature Conservation (ICCN)
	Mozambique National Consortium	Dr Luis Neves, Faculty of Veterinary Medicine, Eduardo Mondlane University, Mozambique	Faculty of Medicine - Eduardo Mondlane University (FM-EMU) Faculty of Veterinary Medicine – Eduardo Mondlane University (FVM-EMU) Directorate of Animal Sciences – Institute of Agricultural Research of Mozambique - Ministry of Agriculture (DCA-IIAM) National Health Institute – Ministry of Health (INS) National Institute for Fisheries Inspection (INIP)
	Zambia National Consortium	Dr. Aaron S. Mweene, Dean, School of Veterinary Medicine, University of Zambia	School of Veterinary Medicine, University of Zambia School of Medicine - University of Zambia Central Veterinary Research Institute (CVRI) Tropical Diseases Research Institute (TDRC)
	South African Institutes in the SACIDS Consortium	Professor Antony Musoke, Director, Onderstepoort Veterinary Institute of the Agricultural Research Council (ARC-OVI), Pretoria, South Africa	National Institute for Communicable Diseases of the National Health Laboratory Service (NICD/NHLS), Johannesburg, South Africa Onderstepoort Veterinary Institute of the Agricultural Research Council (ARC-OVI), Pretoria Faculty of Veterinary Science University of Pretoria (FVS-UP), at Onderstepoort Stellenbosch University, Medical School, Cape Town
**London strategic smart partners**	London International Development Centre, University of London Royal Veterinary College, University of London London School of Hygiene and Tropical Medicine, University of London Institute of Education, University of London Imperial College, London Institute for Animal Health		
**South-South collaborating institutions**	East African Integrated Disease Surveillance Network (EAIDSNet) Faculty of Tropical Medicine and Center of Excellence for Biomedical and Public Health Informatics (BIOPHICS), Mahidol University, Bangkok, Thailand Southern African Development Community (SADC) Epidemiology and Informatics Sub-committee of the Livestock Technical Committee SADC Trans-Boundary Animal Diseases (TADs) Programme of SADC Secretariat African Field Epidemiology Network (AFENET) African Research Consortium for Ecosystems and Population Health (AFRIQUE One) Southern African Consortium for Research Excellence (SACORE) Connecting Organizations for Regional Disease Surveillance (CORDS) Mekong Basin Disease Surveillance (MBDS) Network		
**Consultative Group on International Agricultural Research (CGIAR) partner**	International Livestock Research Institute (ILRI), Nairobi, Kenya		
**Other collaborating institutions from the North**	Centre for Population and Eco-Health, University of Glasgow, United Kingdom Centre for Infectious Diseases, University of Edinburgh, United Kingdom Global Health and Security Initiative of the Nuclear Threat Initiative (NTI), Washington, D.C., United States Centre for Zoonosis Control Hokkaido University, Japan School of Veterinary Medicine, University of Calgary, Canada Department of Geography, University of Cambridge, United Kingdom International Institute for Environment and Development, London, United Kingdom Institute of Tropical Medicine and International Health, Berlin, Germany Meteorology Office, Hadley Centre, Exeter, United Kingdom Fondation Mérieux, France InSTEDD (Innovative Support to Emergencies, Diseases and Disasters), Stanford University, California, USA and Cambodia		

SACIDS seeks to enhance the effectiveness of existing official disease surveillance systems. Thus, at both national and regional levels, SACIDS is underpinned by sector ministries and regional inter-governmental organizations, especially SADC, the New Partnership for Africa's Development (NEPAD), and the African Union. At the national level, each NatCIDS includes as active members representatives of the Ministries responsible for human health, livestock and wild animal health; operates under the patronage of national chief medical and veterinary officers; and is linked to national offices responsible for responding to natural emergencies. At the regional, or SADC level, SACIDS is developing linkages with sections of the SADC Secretariat that deal with human and animal health matters, not only through desk officers but also directly with the communicable diseases and livestock technical committees that advise governments on priority diseases for regional priority.

## Activities and Achievements

**Fig. 1:**
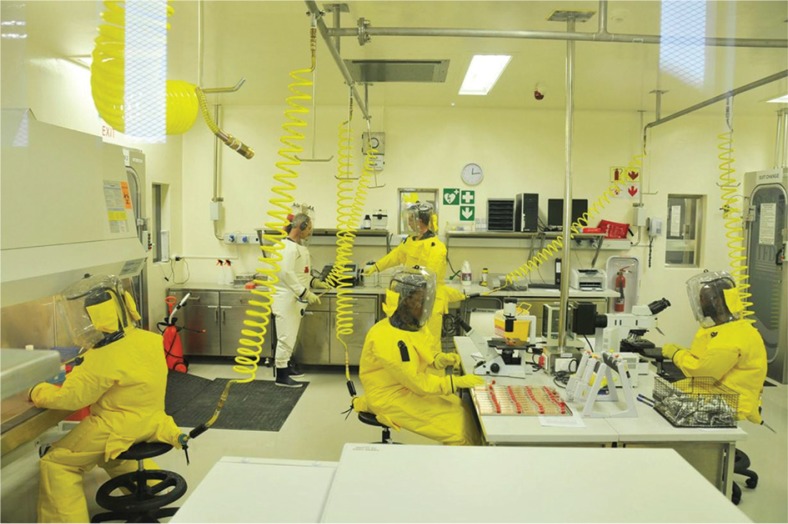
Scientists from the SADC region, including a SACIDS-sponsored postdoctoral research fellow, working in the only biosafety level 4 (BSL-4) laboratory in Africa, which is located at the National Institute for Communicable Diseases, Johannesburg, South Africa. Source: SACIDS.

The initial focus of SACIDS is on capacity development through employment of the Community of Practice (CoP) principle (19). A CoP is a participatory partnership among people who share a common concern and interest, in this case the vision and mission of SACIDS, and who convene regularly to learn from each other (20). Building on existing strengths and programs, SACIDS is focused on four major sets of activities:

1. Provide training through two “One Health” MSc courses, one at Sokoine University, Tanzania, with a focus on molecular biology; the other at the University of Zambia, Lusaka, with a focus on analytical epidemiology. Each course includes core modules on the understanding of key One Health challenges. The courses have been developed and are being delivered in collaboration with regional institutions and the University of London's London School of Hygiene and Tropical Medicine (LSHTM) and Royal Veterinary College (RVC).
2. Develop research capacity, focusing primarily on five disease-driven themes: (i) climate-dependent, vector-borne diseases (e.g., Rift Valley fever); (ii) diseases with potential inter-species concern/spread between wildlife, livestock, and humans (e.g., tuberculosis); (iii) diseases of economic and food security importance (e.g., foot-and-mouth disease); (iv) bacterial rare diseases (e.g., plague); and (v) dangerous emerging diseases (e.g., viral haemorrhagic fevers). The consortium's research on One Health policy is focused primarily on disease burden in the dry land ecosystems of southern and East Africa and its impact on livestock-dependent communities (21). Another cross-cutting area of research is that on participatory epidemiology (22) and the use of mobile technologies to collect and transmit field data.
3. Share expertise and resources across consortium institutions, especially for disease emergency situations (See Figure. 1). The value of this approach has been demonstrated by the discovery of two new arenaviruses, LuJo (23, 24) and Luna (25).
4. Examine approaches and mobile technologies for improving the efficiency of disease alerts, surveillance and response (26).

**Fig. 2:**
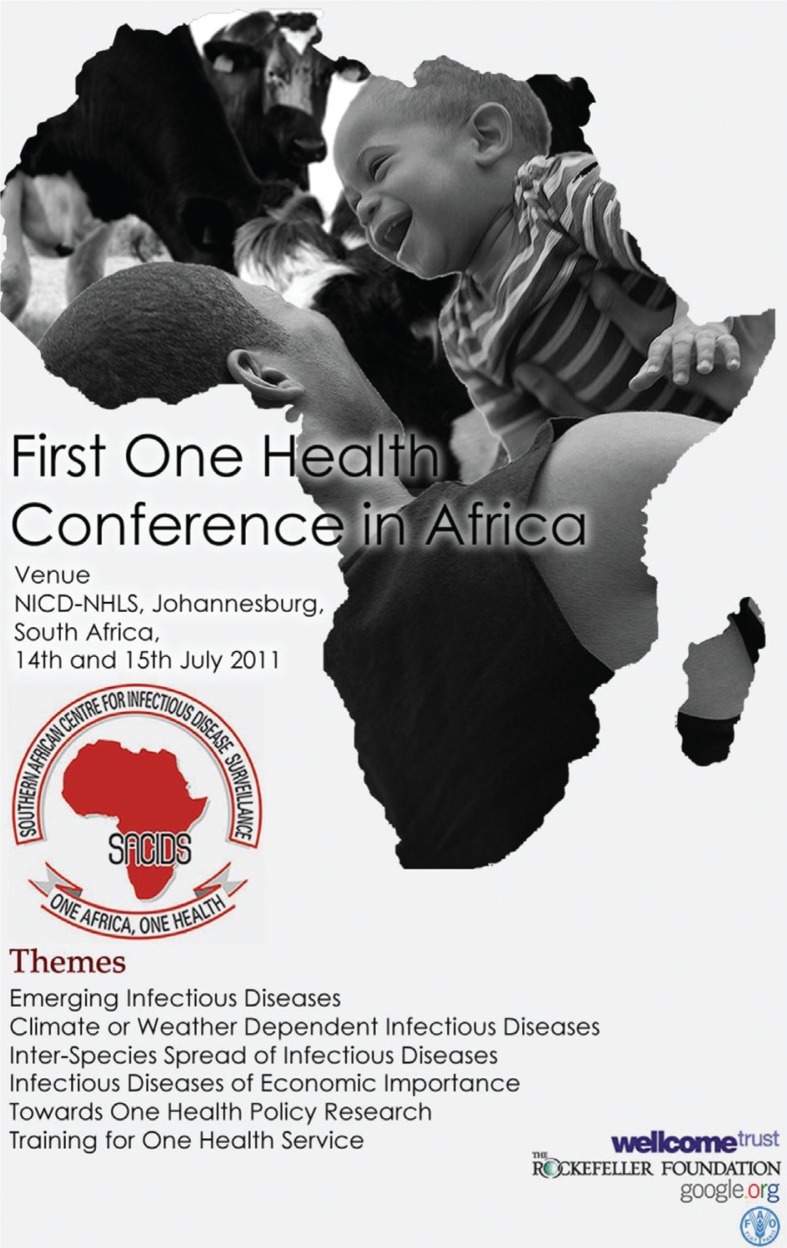
A poster for the first One Health conference held in Africa, in July 2011, which was convened by SACIDS. Source: SACIDS.

Additionally, SACIDS organized the first One Health conference in Africa at the National Institute for Communicable Diseases, Johannesburg, South Africa, in July 2011 (Figure. 2). The conference covered the same research themes listed above (under the second bullet point). For each theme, young scientists presented a series of short papers, followed by a keynote paper by an invited specialist of international repute. The final session was addressed by the special conference guest, Dr. David Nabarro of the United Nations, who spoke about ways that the One Health approach is contributing not just to health but also to food security and community economic well-being. Nabarro emphasized the importance of overcoming the tendency to work in “professional niches and bureaucratic silos and, instead, sharing data and analyses, developing joint policies, doing research together, implementing joint investigations and being accountable for delivering results” (27). Nabarro's address was followed by a grand debate by invited specialists who described the various facets of One Health. The conference proceedings were published in a special supplement of *Onderstepoort Journal of Veterinary Research* (28).

SACIDS One Health efforts complement other efforts to integrate human and animal disease surveillance. For example, in the spirit of One Health, the Tanzania Field Epidemiology and Laboratory Training Programme (FELTP) is governed by a multi-sectoral steering committee whose members include representatives from the Ministry of Livestock Development and Fisheries, and efforts are underway to establish a veterinary stream of the Tanzania FELTP. TFELTP is a collaboration between MOHSW, Muhimbili University of Health and Allied Sciences (MUHAS), National Institute of Medical Research (NIMR), Centres for Disease Control and Prevention (CDC), World Health Organization (WHO), and African Field Epidemiology Network (AFENET) (25). It was established by the Ministry of Health and Social Welfare (MOHSW) in 2008, following an assessment of Tanzania's existing public health and surveillance systems and recognition of the need for a competently trained public health workforce (29). Additionally, most SADC countries have adopted the WHO-AFRO system for Integrated Disease Surveillance and Response (IDSR), which promotes a One Health-based strategy (30).

## Case Studies

Text Boxes 1 and 2 present case studies illustrating the ongoing activities and early accomplishments of SACIDS. Text Box 1 describes how SACIDS's selection of tuberculosis (TB) as a priority disease reflects not only SACIDS's focus on One Health, given the potential spread of TB between animals and humans, but also seeks to enhance the effectiveness of already existing surveillance systems.

***Text Box 1.* Tuberculosis as a priority disease for both SACIDS and SADC**

Various *Mycobacteria* strains, the causative agent(s) of tuberculosis (TB), can infect both animals and humans. SACIDS's focus on TB exemplifies not only how SACIDS is focused on developing and implementing a One Health approach to infectious disease surveillance, but also how SACIDS seeks to enhance existing surveillance systems by prioritizing the same diseases that the official organs of SADC prioritize. SADC prioritizes TB because member states carry a disproportionate burden of the dual epidemic of TB and HIV/AIDS compared to the rest of Africa and the rest of the world. The region is home to 25 percent of the sub-Saharan human population but accounts for 50 percent of TB cases reported. The SADC Health Protocol includes a specific Article 12 on TB control, advocating for global and regional partnerships to respond to the TB epidemic in the SADC region (30). This fact, coupled with a lack of knowledge about the role of zoonotic *Mycobacteria* strains in the infection of humans, was a key justification for the selection of TB as an exemplar disease for study by SACIDS.

***Text Box 2.* Piloting mobile technologies and One Health surveillance approaches**

**Fig. 3:**
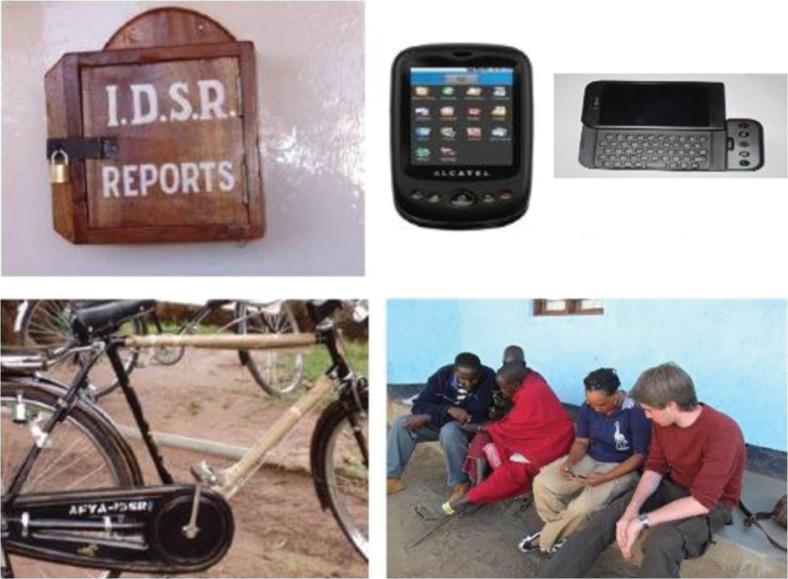
Images of the SACIDS-EAIDSNet piloting mobile technology being used to conduct One Health disease surveillance in Ngorongoro (Tanzania), the Kagera Basin (cross-border across Rwanda-Burundi-Tanzania), and the Zambezi Basin (Zambia) ecosystems. Source: SACIDS.

With funding from the Rockefeller Foundation, SACIDS is collaborating with EAIDSNet on the pilot application of a One Health-based mobile technology approach to disease surveillance. The project operates in Tanzania, Zambia, and Burundi. In Tanzania, the approach has been to involve human, livestock, and wildlife sectors at the national and local levels, relying on specialists from both the human and animal sectors to agree on a set of target diseases and to design shared data-collection forms. The forms are programmed into Android-driven mobile telephones using the EpiCollect and ODK programmes (31). Primary health workers enter disease data into the mobile telephones; and data are transmitted via the mobile telephone network to a server at SACIDS headquarters for storage, analysis, and mapping (Fig. 3). Piloting efforts thus far have shown that for effectiveness and sustainability, a mobile technology-based disease surveillance system will require three key elements: i) participatory epidemiological approaches; ii) form-based reporting; and iii) resident ICT expertise for programming at the discovery end and for local support, database handling, customized programming, trouble-shooting, and training at the user end (22).

The case study described in Text Box 2 demonstrates how SACIDS is fostering inter-sectoral collaboration in One Health surveillance and response through the use of mobile technologies. Once rolled out, the One Health-based mobile technology system described in Text Box 2 will help to meet the need for a systemic exchange of disease surveillance data across the human and animal health sectors within SADC. There is no such exchange occurring except via vertical programs like the ongoing WHO-supported rabies elimination project in Tanzania and South Africa. The episodes of Rift Valley fever in 1997/8 and 2007 in Tanzania brought to the fore the need for such exchange (16, 28).

## Relationship to CORDS

CORDS provides a common vision and goal for disease surveillance that transcends regions; allows for South-South-North exchange of experiences and mutual trust; and enables bilateral collaboration between disease surveillance networks from different regions and even different parts of the world (29). SACIDS stands not only to benefit CORDS, but was itself a builder of CORDS. SACIDS participated in all of the key meetings on regional disease surveillance that led up to the formation of CORDS in 2011 and is a founding member of CORDS (16). An example of the collaborative effort made possible by SACIDS's participation in CORDS is the joint SACIDS-EAIDSNet exploration of mobile technologies for disease alerts and surveillance in remote and cross-border areas (Text Box 2).

## Challenges and Way Forward

The southern and East African regions suffer from among the highest animal and human infectious disease burdens in the world. The future will likely see a growing number of infectious disease outbreaks among both animals and humans as a result of climate change, interventions themselves (e.g., new vaccines), pathogen evolution, travel and trade, changing patterns of land use resulting in increased interactions between humans and both domestic and wild animals, increasing urbanization, population growth, and changing food consumption patterns. Together, these factors will create evolving One Health challenges, such as emerging zoonoses, and an increasing demand for scientific evidence in relevant policy decision-making. The challenges will be made more difficult if policy silos between human health, animal health, and agriculture prevent the type of inter-sectoral, inter-disciplinary collaboration that is needed for One Health infectious disease surveillance and response.

But as the burden increases, so too do opportunities for reducing the burden. It is anticipated that SACIDS itself will continue to evolve as an initiative that promotes novel One Health approaches to infectious disease surveillance, such as the application of improved diagnostic and information technologies that can be used in remote rural settings. To be effective, SACIDS will need to strengthen its “engagement” and convening strategy for developing smart, shared-vision partnerships at national, regional, and international levels. CORDS helps to nurture that strength.
